# Notes of Hospital Clinics

**Published:** 1897-08-28

**Authors:** 


### Medical Progress and Hospital Clinics.

[*The Editor will be glad to receive offers of co-operation and contributions from members of the profession. All letters should be addressed to* The Editor, at the Office, 28 & 29, Southampton Street, Strand, London, W.C.]

### Notes of Hospital Clinics.

[*Reported for* The Hospital, *and corrected by the authors.*]

### ST. BARTHOLOMEW'S HOSPITAL.

On a Case of Tetanus. A Portion of a Clinical Lecture.

By Howard Marsh, F.R.C.S., Surgeon to the Hospital.

Gentlemen,—A man, aged 52, was recently admitted into St. Bartholomew's Hospital, in whom the symptoms of tetanus had been developed eight days previously. On his admission he had well-marked trismus, and an equally well-marked risus sardonicus; the abdominal muscles were hard and rigid, and there was some stiffness of the lower limbs. His symptoms were reported to be increasing. There was no evidence to show at what point inoculation with the tetanus bacillus had occurred. There were numerous superficial abrasions of his hands, but there was no definite wound anywhere about him. He was able to get fluid food between his teeth, and to swallow a fair amount. His pulse was about 90, and of good volume. He had no severe spasms, and he complained of no material pain.

The symptoms indicated in a conclusive manner that the disease was true tetanus, but as the patient had been already suffering for eight days, and as his symptoms were now of only moderate severity the case evidently did not belong to the acute class, but rather to that less severe form in which the prognosis is always comparatively hopeful. In the absence of an obvious centre of local inflammation indicating the place of inoculation, the case might, according to the old classification, have been called "idiopathic." In our present state of knowledge, however, as to the pathology of tetanus, the term "idiopathic" seems absolutely without meaning. One might as well speak of a case of idiopathic scarlet fever or idiopathic syphilis. In tetanus, equally with the two diseases mentioned, the symptoms are produced invariably by a specific cause, and in every case of tetanus alike, whether it be acute or chronic in its course, there must have been a specific inoculation. In other words, the cause of tetanus in the chronic is precisely the same as it is in the acute form of the disease. There must be an inoculation with the tetanus bacillus. Doubtless the phrase "idiopathic tetanus" will soon be eliminated from our nomenclature.

As we were unable to recognise the point of inoculation, no local treatment could be adopted. Had the seat of inoculation been apparent, and had it been situated in one of the fingers or toes, amputation would have been at once performed; or if the inoculation wound had involved the soft parts elsewhere, the area would have been freely excised. All observers agree that the tetanus bacillus is met with only in the immediate neighbourhood of the wound of inoculation, and that it is here that the specific poison is developed and hence absorbed and distributed. As this is the case, amputation or excision has the effect of cutting off any further supply, and is, therefore, a proceeding which contributes very largely to the arrest of the disease.

As soon as possible after the patient's admission, he was treated by injection of the tetanus anti-toxin prepared by Professors Tizzoni and Cattani. The dose used was 5 cc. of a solution, equal to half a gramme of Tizzoni's dried serum. The next morning the patient seemed better. There was no increase in any of the symptoms; but, on the contrary, all had somewhat diminished in severity. He had slept fairly well. He took sufficient nourishment in a fluid form, and his pulse was of good volume and under 90. The bowels acted freely after an aperient. For the next seven days the injections were repeated (the same dose being used) once a day, so that he had in all eight injections. These were made into the subcutaneous tissue of the abdominal wall. They produced no local ill effect. The patient steadily improved. The risus and trismus became less and less marked, and the stiffness of the lower limbs passed off, and we were able to regard him as quite out of danger on about the fifth day after the treatment was begun. The last symptom to disappear, and this persisted for nearly a fortnight, was the rigidity of the muscles of the abdominal wall. This persistency of rigidity of these muscles after all other active symptoms have passed off has been noticed in other cases.

As I have already said, this was obviously a case of true tetanus, although it was of a mild rather than a severe type. When the patient was admitted he was steadily getting worse, but as soon as the treatment I have described was commenced improvement set in, and it steadily continued until his recovery was complete. The experience hitherto obtained in the treatment of tetanus by anti-toxin injections is limited, and therefore each case is of value. In this instance it appeared to those who watched the case quite clear that the disease was controlled and finally cured by the injections.

### KING'S COLLEGE HOSPITAL.

Clinical Lecture on Three Cases of Enlarged Spleen. Delivered on July 10, 1897.

By Professor Curnow, M.D., F.R.C.P., Physician to the Hospital.

After pointing out the special features of the three cases on which the demonstration was based, Professor Curnow dealt with the general subject under the following heads:—

The diagnosis of an enlarged spleen is usually simple, the vertical diameter of splenic dulness in a normal spleen being three inches, and the transverse diameter two inches.

No growth of the stomach is likely to be mistaken for an enlarged spleen, but impacted fæces in the splenic flexure of the colon might be mistaken for it; therefore, in every case of supposed enlargement of the spleen, always empty the colon before making a diagnosis.

Tumours of the left kidney might be mistaken for splenic tumours, but it should be remembered that most tumours of the kidney are fluid, whereas most tumours of the spleen—with the exception of hydatids —are solid. Fluid in the left pleura might be mistaken for it, and so might a solid effusion into—or a growth implicating—the left lung.

Fortunately for the diagnosis, splenic tumours grow mainly downwards and forwards; but when the spleen is not very large there may be doubt. The spleen moves up and down with the diaphragm with respiration; so to aid the diagnosis you should have the patient standing up, and put your fingers under the costal margin on the left side, and make him or her breathe. Feeling the notch of the spleen is supposed to settle the diagnosis; but remember that the notch cannot always be felt, for the edge of the spleen sometimes is hidden by the tendency of the viscus when enlarged to have its anterior margin bent backwards away from the abdominal wall.

The main points to be noticed in these true uncomplicated splenic cases are: (1) There is usually fever, which is generally of the intermittent type. (2) There may be symptoms of pressure on the lumbar plexus, and even on adjacent viscera. In one case the tumour pressed on the sacral plexus, causing intense sciatic pain. (3) The blood changes. Patients are markedly anæmic, and there is a great tendency to epistaxis and bleeding from the gums, in addition to special microscopic changes on examination of the corpuscles.

I will now refer to some of the different kinds of enlargement that the spleen may undergo, and discuss them seriatim.

1. It undergoes slight physiological distension and contraction; and there is a slight physiological enlargement after a large meal.

2. It enlarges in every acute infectious disease, *e.g.*, in enteric fever, septic poisoning, ulcerative endocarditis.

3. In cases of valvular disease of the heart, pain and local swelling due to emboli are often present.

4. Ague cake. This is a chronic fibroid enlargement sequential to a chronic congestion. In all acute cases the blood should be examined for the plasmodium of ague. No plasmodium was found in the acute case, who had subsequently died from septicæmia.

5. Chronic congestion due to chronic cardiac or hepatic disease. When there is not much enlargement of the spleen there is much ascites, and *vice versa*, because the blood is stored up in the spleen instead of exuding its serum into the peritoneum.

6. Growths and degenerations. (*a*) Leukemia: This disease consists of the blood change plus the enlargement of the spleen. The enlargement of the spleen here is a true hypertrophic change—all the elements of the spleen being enlarged. (*b*) Lymphadenoma: This consists of the blood change plus enlargement of the glands. (*c*) Lympho-sarcoma: In this disease the spleen is enlarged, but there is no blood change. Mixed cases of (*a*) and (*b*) sometimes occur. True cancer and sarcoma are so rare that they may be left out of consideration.

7. Lardaceous degeneration. This is very common in connection with prolonged suppuration, with phthisis, with rickets, syphilis, and other constitutional affections.

8. Tuberculous spleen usually occurs in connection with tuberculosis of other organs.

9. Acute syphilitic enlargement occurs occasionally during the febrile stage, when the sore throat and rash are present. It occurs in congenital or late syphilis. It may be a peri-splenitis, *i.e.*, inflammation of the fibrous capsule of the spleen; or, more generally, proliferation of fibrous tissue, which later leaves a hard cirrhotic spleen. Gummata in the spleen are rare.

10. Hydatid cyst in the spleen is rare, but may be mistaken for one in the left lobe of the liver.

---

### THE ADMINISTRATION OF CHLOROFORM BY REGULATING INHALER.

By J. O. Symes, M.D.Lond., Anæsthetist to the Royal Hospital for Children, Bristol.

The question whether the fatal accidents from chloroform are the result of over-dosage or of an idiosyncrasy in the patient is one which is still warmly debated. Were the use of regulating inhalers more common, data would soon be obtained affording valuable information upon this subject. By means of Junker's apparatus the operator is enabled to regulate the dose of chloroform with great exactness, and at the same time to ensure its admixture with a proper amount of air. More than this, he is able, at any moment, to state what quantity of chloroform the patient has inhaled, and at what rate he got it, two items of information which would be invaluable in inquiring into a fatal case of chloroform narcosis. It has been claimed that such accidents do not occur when the drug is administered by means of a regulating inhaler, but a very much wider use of the apparatus is needed before such a statement can be substantiated. It certainly would seem probable that the open method of administration, with its varying quantities of chloroform and air, is riskier, although even here one must distinguish between the rough and ready method of pouring chloroform on a towel or roll of lint and the more exact method with drop-bottle and Skinner's mask. Apart altogether from the question of safety, the use of Junker's inhaler, or of one of its modifications, presents several marked advantages.

In the first place the time occupied in inducing that degree of narcotism necessary for the painless performance of the operation is much shortened. Using a drop bottle and Skinner's mask this period occupies from eight to ten minutes, whilst with an inhaler it does not exceed five. At the present time when surgeons spend so much time and care in sterilising the skin, any safe method of shortening the period during which the patient is under the influence of the drug is welcome. With the inhaler, too, the operator has much greater command over the patient, a few extra compressions of the bellows sufficing at any time to deepen the anæsthesia. The economy in chloroform is very great, the quantity used being less than half that required when the open method is used. This is particularly noticeable in hot weather. The evaporation from a mask at such a season is often so great as to render it exceedingly difficult to keep the patient under the anæsthetic, but with Junker's apparatus little or no difference is to be detected. With this method also the operator is spared the discomfort of constantly breathing the drug himself. He knows that instead of it being shared, as in the open method, between the patient, the operator, and the atmosphere of the room, the chloroform is administered to the patient only. I have not found that children are frightened by the mask, nor do they seem to experience any sense of suffocation when it is firmly applied to the face. There is a tendency, however, for them to fall asleep more quickly under this method, and this as far as possible should be prevented.

For the purpose of comparison I have taken notes of two series of cases, in one of which chloroform was administered by the open method, whilst in the other Junker's apparatus was used. Each series consists of 20 cases. Space will not permit me to give details of these 40 cases.

In the first series the average age of the children was a little under five years. Four thousand eight hundred and sixty minims of chloroform were administered in 687 minutes, the average amount per minute being thus seven minims. The average temperature of the room was 63·1 deg. F.

In the second series in which Junker's apparatus was used the average age of the patients was between five and six years. Two thousand seven hundred minims of chloroform were given in 895 minutes, the average amount per minute being three minims. The temperature of the room averaged 72·9 deg. F., the season being the end of July and beginning of August. It may be of interest to give the details of a few of the cases belonging to the second series:—

| Sex. | Age. | Quantity of chloroform in minims. | Time occupied in administration. | Aver. amount per minute in minims. | Temperature. |
|---|---|---|---|---|---|
| M.... | 4 days. ... | 90 ... | 35 ... | 2·5 ... | 67° F. |
| M.... | 8 yrs. ... | 360 ... | 140 ... | 2·5 ... | 66 |
| F. ... | 6 mths. ... | 45 ... | 20 ... | 2·2 ... | 75 |
| M.... | 7 yrs. ... | 30 ... | 7 ... | 4·2 ... | 75 |
| F. ... | 10 yrs. ... | 270 ... | 105 ... | 2·5 ... | 65 |
| F. ... | 8 yrs. ... | 240 ... | 60 ... | 4 ... | 68 |
| M.... | 4 yrs. ... | 240 ... | 75 ... | 3·2 ... | 72 |
| M.... | 10 yrs. ... | 150 ... | 30 ... | 5 ... | 75 |
| F. ... | 6 mths. ... | 60 ... | 30 ... | 2 ... | 81 |

These figures show that, although the age of the patient was slightly greater, and the temperature of the room decidedly higher, in the series in which a regulating inhaler was employed, yet anæsthetisation was accomplished and maintained with the expenditure of less than one-half of the chloroform required by the open method. The face-piece employed was of the original Junker type for adults; but with a child's face-piece of a modified form, such as Krohne and Sesemann's, or Dudley Buxton's, a still smaller quantity of chloroform would have been required. Apart, then, from the question of safety, this method of administration has, with children, the advantages of being speedy and easily controlled. It is, too, unaffected by temperature, and is conducted with the minimum of discomfort to the operator. These reasons alone should be sufficient to ensure it being more widely adopted than is at present the case.

